# *Pseudomonas rhizophila* S211, a New Plant Growth-Promoting Rhizobacterium with Potential in Pesticide-Bioremediation

**DOI:** 10.3389/fmicb.2018.00034

**Published:** 2018-02-23

**Authors:** Wafa Hassen, Mohamed Neifar, Hanene Cherif, Afef Najjari, Habib Chouchane, Rim C. Driouich, Asma Salah, Fatma Naili, Amor Mosbah, Yasmine Souissi, Noura Raddadi, Hadda I. Ouzari, Fabio Fava, Ameur Cherif

**Affiliations:** ^1^Univ. Manouba, ISBST, BVBGR-LR11ES31, Biotechpole of Sidi Thabet, Ariana, Tunisia; ^2^Laboratory of Microorganisms and Active Biomolecules, MBA-LR03ES03, Faculty of Sciences of Tunis, University of Tunis El Manar, Tunis, Tunisia; ^3^Department of Civil, Chemical, Environmental and Materials Engineering (DICAM), University of Bologna, Bologna, Italy

**Keywords:** *Pseudomonas rhizophila*, plant-growth promotion rhizobacterium, biofertilization, phytostimulator, pesticide bioremediation, biosurfactant production

## Abstract

A number of *Pseudomonas* strains function as inoculants for biocontrol, biofertilization, and phytostimulation, avoiding the use of pesticides and chemical fertilizers. Here, we present a new metabolically versatile plant growth-promoting rhizobacterium, *Pseudomonas rhizophila* S211, isolated from a pesticide contaminated artichoke field that shows biofertilization, biocontrol and bioremediation potentialities. The S211 genome was sequenced, annotated and key genomic elements related to plant growth promotion and biosurfactant (BS) synthesis were elucidated. S211 genome comprises 5,948,515 bp with 60.4% G+C content, 5306 coding genes and 215 RNA genes. The genome sequence analysis confirmed the presence of genes involved in plant-growth promoting and remediation activities such as the synthesis of ACC deaminase, putative dioxygenases, auxin, pyroverdin, exopolysaccharide levan and rhamnolipid BS. BS production by *P. rhizophila* S211 grown on olive mill wastewater based media was effectively optimized using a central-composite experimental design and response surface methodology (RSM). The optimum conditions for maximum BS production yield (720.80 ± 55.90 mg/L) were: 0.5% (v/v) inoculum size, 15% (v/v) olive oil mill wastewater (OMWW) and 40°C incubation temperature at pH 6.0 for 8 days incubation period. Biochemical and structural characterization of S211 BS by chromatography and spectroscopy studies suggested the glycolipid nature of the BS. *P. rhizophila* rhamnolipid was stable over a wide range of temperature (40–90°C), pH (6–10), and salt concentration (up to 300 mM NaCl). Due to its low-cost production, emulsification activities and high performance in solubilization enhancement of chemical pesticides, the indigenous BS-producing PGPR S211 could be used as a promising agent for environmental bioremediation of pesticide-contaminated agricultural soils.

## Introduction

Overuse of chemical fertilizers and pesticides to meet the growing demand of food supply has undoubtedly cause pollution and severe damage to soil organisms and insect pollinators (Savci, 2012; Mahanty et al., 2017). Nonetheless, the outcome of using excess chemical inputs has made the crops more susceptible to diseases and decreased soil fertility (Tilman et al., 2002; Aktar et al., 2009). Considering the hazardous effects of chemical fertilizers, biofertilizers are supposed to be a safe alternative to chemical inputs and minimizes ecological disturbance to a great extent. Biofertilizers are ecofriendly agro-input, more cost-effective than chemical fertilizers, and their prolonged use enhances soil fertility substantially (Mahdi et al., 2010; Singh et al., 2011). It was mentioned that the use of biofertilizers enhances crop yield by 10–40% (Bhardwaj et al., 2014). The use of biofertilizers has many benefits, including cheap source of nutrients, excellent suppliers of micronutrients and organic matter, secretion of growth hormones, no adverse effects to ecosystem and longer shelf life (Gaur, 2010; Mahanty et al., 2017).

Plant growth promoting rhizobacteria (PGPR) are the soil bacteria that colonize the root surface and promote plant growth via secretion of regulatory chemicals in the vicinity of the rhizosphere (Cherif et al., 2017). PGPR enhance the plant growth directly by facilitating water and nutrient uptake and modulating phytohormone levels, or indirectly by inhibiting pathogens in the forms of biocontrol agents (Ahemad and Kibret, 2014). Besides offering economically and ecologically attractive means for increasing the nutrient supply and protecting against soil borne pathogens, PGPR are the key players in efforts to alleviate plan stress and to enhance bioremediation of polluted soils (Zhuang et al., 2007; El-Daim and Moustafa, 2015). In fact, BS-producing PGPR capable of solubilizing and degrading certain kind of pollutants (i.e., explosives, herbicides or hydrocarbons) have been isolated from different contaminated sites and the potential degradation pathways, and enzyme-encoding genes have been studied (Burd et al., 2000; Germaine et al., 2006; Sheng et al., 2008; Kruijt et al., 2009; Singh and Cameotra, 2013).

Microbial BS include a large number of chemical structures, such as lipopeptides, phospholipids, glycolipids, and polymeric macromolecules like exopolysaccharides (Mukherjee et al., 2006; Jadhav et al., 2013). Recently, there has been considerable interest in the properties of microbial BS such as biodegradability, low toxicity, biocompatibility, digestibility, diversity for chemical structure and activities, and effectiveness even at extreme conditions including heat, alkalinity and salinity (Fakruddin, 2012; Sarubbo et al., 2015a,b). BS are expected to reach more than 2 billion USD by 2020, with different bioremediation applications such as enhanced oil recovery, heavy metals removal, and pesticides detoxification in contaminated sites (Maier, 2003; Ying, 2006; Franzetti et al., 2009; Pacwa-Płociniczak et al., 2011; Bustamante et al., 2012; Sarubbo et al., 2015b).

Currently, one of the major problems in the production of microbial BS at a large-scale is the high production costs (Geys et al., 2014). In order to make it economically competitive, it is necessary to reduce substrate cost, optimize culture conditions using experimental designs, improve recovery process, and use overproducing strains for high yields. As fermentation medium can represent about 30% of the production cost, BS can be produced from inexpensive waste substrates, thereby dramatically reducing the microbial culture cost (Sarubbo et al., 2015a). Olive oil mill wastewater (OMWW) is a phenolic-rich industrial effluent that can be considered as a serious environmental problem, particularly in olive oil producing Mediterranean countries. The high content of polyphenols in OMWW is problematic for biological processing. However, the presence of polysaccharides, free sugars and residual oil, suggest that OMWW could be advantageously valorized and used as a carbon source for microbial biosurfactant (BS) production (Colak and Kahraman, 2013; Ramírez et al., 2015; Neifar et al., 2017).

The present study investigates the ability of a novel species of *Pseudomonas*, isolated from pesticide-contaminated artichoke farm soil, to promote plant growth and to produce BS. The *in vitro* tests and the genome sequencing, assembly and annotation revealed that the S211 strain has a wide spectrum of PGP traits including biocontrol, biofertilization and rhizoremediation activities. This study also describes the statistical optimization of BS production, the biochemical characterization and structural identification of BS from S211, as well as the evaluation of its ability to enhance pesticide solubilization. Further characterization of multi-trait PGPR such as *P. rhizophila* S211 will be promising to design an effective strategy for a sustainable agriculture development.

## Materials and methods

### Reagents and chemicals

All chemicals and reagents used in this study were of pure analytical-grade and available commercially.

### Bacterial isolation and PGP activities

Rhizospheric soil sample was collected from pesticides-contaminated field in Sidi Thabet, an agricultural region of northern Tunisia “36°54′31.1″N; 10°2′32.89″E.” Bacterium isolation was carried out by an enrichment culture technique in mineral salt medium (MSM) supplemented with 100 mg/L of dimethoate as a sole carbon source. MSM medium contained the following components at the specified concentrations (in g/L): (NH_4_)_2_SO_4_, 2; MgSO_4_ 7H_2_O, 0.2; CaCl_2_ 2H_2_O, 0.01; FeSO_4_ 7H_2_O, 0.001; Na_2_HPO_4_ 12H_2_O, 1.5; KH_2_PO_4_, 1.5; pH8 (Cycon et al., 2009). Growth of S211 strain was performed in Nutrient Agar (NA) at different pH values of 7, 9, and 11. The same culture medium supplemented with 0, 5, 10, and 20% of NaCl (w/v) was used to test the growth behavior of the strain after 3 days of incubation at 30°C. S211 isolate was screened *in vitro* for various plant growth promoting properties. Screening for N_2_-fixing activity of the pure bacterial culture was determined on Jensen's N-free medium as reported by Jensen (1942). The ability of the isolate to solubilize the inorganic tricalcium phosphate [Ca_3_ (PO4)_2_] was checked on to the National Botanical Research Institute's medium Phosphate (NBRIP) according to the method of Nautiyal (1999). Siderophore production ability of S211 was determined in CAS agar medium (Chrome Azurol S medium) as described by Alexander and Zuberer (1991). Indole Acetic Acid (IAA) production was carried out using Salkowaski's method (Gordon and Weber, 1951). Production of ammonia (NH3) by S211 was qualitatively tested as reported by Cappuccino and Sherman (1992). The method described by Alström and Burns (1989) was adopted to evaluate the ability of S211 strain to produce hydrogen cyanide (HCN). Siderophores-pyoverdine was detected in King's B (KB), succinate medium (SM), and Casamino Acid media (CAA) according to the method of Page and Tingerstrom (1988). The inhibitory power between the bacterial strain (*P. aeruginosa* ATCC27853) and the pyoverdine extract was checked in TSA. S211 strain was also screened for their EPS-producing potential as described by Vijayabaskar et al. (2011). The assay plate used for the detection of anionic-BS was performed in solid MSM medium supplemented with OMWW as a sole carbon source, cetyltrimethyl ammonium bromide (CTAB) and methylene blue (MB) (Satpute et al., 2008). The oil displacement activity was determined according to Techaoei et al. (2011).

### S211 genome sequencing, assembly, and annotation

Genomic DNA extraction was performed using the MagNA Pure LC DNA isolation KitIII (Roche) and was sent to Inqaba Biotechnical Industries, a commercial NGS service provider, for whole genome sequencing. Briefly, genomic DNA sample were fragmented using an ultrasonication approach (Covaris), size-selected and end repaired. Each generated fragment was ligated to illumina specific adapter sequence, quantified, indexed, size selected (AMPure XP Bead-based) and then sequenced on illumina's MiSeq platform, using a MiSeq v3 (600 cycle) kit. 250 Mb of data (2 × 300 bp long paired end reads) were produced for each sample. Quality of sequence reads was first analyzed using the FastQC tool (Andrews, 2011). Then, adaptor sequence removal, trimming, error correction, and assembly were performed using the A5-miseq pipeline, an integrated pipeline for de novo assembly of microbial genomes (Tritt et al., 2012), and finally analyzed with QUAST (Gurevich et al., 2013). These contigs were finally ordered using CONTIGuator v2.3 (Galardini et al., 2011) with its closely related genomes, *P. lini* (NZ_LT629746.1), *P. frederiksbergensis* (NZ_CP017886.1) *P. mandelii JR-1* (NZ_CP005960.1), and *P. brassicacearum* (CP012680.1) using CONTIGuator (Galardini et al., 2011). The assembled genome of *P. rhizophila* S211 consists of 5.98 Mbp distributed over 26 contigs and organized in one scaffold with fold coverage of 80X. Gene predictions and annotations were performed with Rapid Annotations using Subsystems Technology (RAST) database (Aziz et al., 2008), Integrated Microbial Genomes/Expert Review (IMG/ER) (Markowitz et al., 2009) and Prokka version 1.1 (Seemann, 2014), which predicts coding DNA sequence (CDS) using Prodigal (Hyatt et al., 2010). tRNA genes and rRNA genes were predicted by tRNAScan-SE software (Lowe and Eddy, 1997) and RNAmmer, respectively (Lagesen et al., 2007). The scaffolds were searched against the KEGG database to analyze metabolic pathways and gene functions (Kanehisa and Goto, 2000). Glimmer3 was used for the prediction of structural genes (Delcher et al., 2007). The predicted ORFs were annotated by searching against the COG and SEED databases. Functional annotation was performed by searching the National Center for Biotechnology Information (NCBI) protein database and the Kyoto Encyclopedia of Genes and Genomes (KEGG) protein database, the cluster of orthologous groups (COG) (Tatusov et al., 2003) database, and the TIGRfam database (Haft et al., 2003).

### Bacterial identification and phylogenetic analysis

Assessment of phylogenetic affiliation of bacterial isolate was first based on 16S rRNA gene sequence analysis according to the procedure described previously (Guesmi et al., 2013) and then confirmed by whole genome sequence analyses. Identification of closely related strains to S211 was performed based on Basic Local Alignment Search Tool (BLAST) searches. 16S rRNA gene sequences were aligned with T-Coffee (v11.00.8cbe486) using Lalign_pair and slow_pair alignments (Weng et al., 2013). The phylogenetic tree was reconstructed using the bayesian inference method implemented in the MrBayes program (v3.2.3). The number of substitution types was fixed to 6. The standard (4 by 4) model of nucleotide substitution was used, while rates variation across sites was fixed to “invgamma.” Four Markov Chain Monte Carlo (MCMC) chains were run for 10,000 generations, sampling every 10 generations, with the first 250 sampled trees discarded as “burn-in.” Finally, a 50% majority rule consensus tree was constructed. Graphical representation and edition of the phylogenetic tree were performed using iTOL v3 (Letunic and Bork, 2016).

### *In silico* DNA-DNA hybridization and average nucleotide identity

The draft genome of S211 strain was used to evaluate its similarity to closely related species based on (i) *in silico* DNA-DNA hybridization (isDDH) determination, using the Genome-to-Genome Distance Calculator (GGDC) version 2.0 (http://ggdc.dsmz.de/distcalc2.php; Auch et al., 2010; Meier-Kolthoff et al., 2013b). isDDH values were calculated using the recommended formula 2 for draft genome assemblies. The isDDH values between genomes of the same species are above 70% (Auch et al., 2010; Meier-Kolthoff et al., 2013b), and (ii) the estimation of the Average Nucleotide Identity (ANI) using best hits and reciprocal best hits between two genomic datasets as described by Goris et al. (2007). Bacterial strains that exhibited more than 95% ANI should belong to the same species (Rodriguez and Konstantinidis, 2014).

### Statistical optimization of BS production by S211 using OMWW as low-cost substrate

Response surface methodology (RSM) and central composite design (CCD) were applied to study the BS production variables by *P. rhizophila* S211 (Myers et al., 2009). This methodology is suitable for fitting a quadratic surface and optimizing the effective variables with a minimum number of experiments, as well as to analyse the interaction effects between factors (Goupy, 1999; Myers et al., 2009). A CCD with 31 experiments was applied for the optimization of BS production. S211 inoculum was prepared in 50 ml tryptic soy broth (TSB) with overnight incubation at 30°C at 150 rpm. BS production was conducted in 250 ml conical flasks containing 100 ml MSM supplemented with OMWW. The effect of five independent variables; pH (X_1_), temperature (X_2_), OMWW (X_3_), inoculum size (X_4_), and incubation time (X_5_) on the rhamnolipid production yield (Response Y), were evaluated at three levels (Table 1). After each incubation time, an aliquot of 10 ml was taken from each flask; 2 ml was used to estimate the bacterial growth by measuring the OD_600_. Eight milliliter was centrifuged at 10,000 rpm for 15 min to remove bacterial cells and the supernatant was used for BS production yield determination according to the Orcinol assay method (Tuleva et al., 2002). This last was used for the direct assessment of the amount of rhamnolipids in the sample as rhamnose (mg/L). To 100 μl of each sample, 900 μl of a solution containing 0.2% orcinol in concentrated sulfuric acid was added. Samples were heated for 30 min at 80°C, cooled at room temperature then OD_421_ was measured. Control was prepared with distilled water. The BS yields were calculated from a standard curve prepared with L-rhamnose.

**Table 1:** Experimental conditions of the CCD design in coded and natural variables and the corresponding observed and predicted responses.

**No. exp**.	**X_1_**	**X_2_**	**X_3_**	**X_4_**	**X_5_**	**pH**	**Temperature (°C)**	**OMWW (%)**	**Inoculum size (%)**	**Incubation time (days)**	**BS production (mg/L)**	
											**Observed**	**Predicted**
1	−1.0	−1.0	−1.0	−1.0	1.0	6.0	20.0	5.0	0.5	8.0	45.32	44.62
2	1.0	−1.0	−1.0	−1.0	−1.0	10.0	20.0	5.0	0.5	2.0	88.40	89.81
3	−1.0	1.0	−1.0	−1.0	−1.0	6.0	40.0	5.0	0.5	2.0	40.00	39.66
4	1.0	1.0	−1.0	−1.0	1.0	10.0	40.0	5.0	0.5	8.0	114.04	114.66
5	−1.0	−1.0	1.0	−1.0	−1.0	6.0	20.0	15.0	0.5	2.0	27.08	25.34
6	1.0	−1.0	1.0	−1.0	1.0	10.0	20.0	15.0	0.5	8.0	52.40	53.62
7	−1.0	1.0	1.0	−1.0	1.0	6.0	40.0	15.0	0.5	8.0	720.80	721.47
8	1.0	1.0	1.0	−1.0	−1.0	10.0	40.0	15.0	0.5	2.0	88.00	96.58
9	−1.0	−1.0	−1.0	1.0	−1.0	6.0	20.0	5.0	2.5	2.0	28.40	30.67
10	1.0	−1.0	−1.0	1.0	1.0	10.0	20.0	5.0	2.5	8.0	288.40	288.64
11	−1.0	1.0	−1.0	1.0	1.0	6.0	40.0	5.0	2.5	8.0	161.60	160.09
12	1.0	1.0	−1.0	1.0	−1.0	10.0	40.0	5.0	2.5	2.0	73.44	73.04
13	−1.0	−1.0	1.0	1.0	1.0	6.0	20.0	15.0	2.5	8.0	150.40	154.49
14	1.0	−1.0	1.0	1.0	−1.0	10.0	20.0	15.0	2.5	2.0	72.40	77.60
15	−1.0	1.0	1.0	1.0	−1.0	6.0	40.0	15.0	2.5	2.0	51.28	54.72
16	1.0	1.0	1.0	1.0	1.0	10.0	40.0	15.0	2.5	8.0	78.80	78.21
17	−1.0	0.0	0.0	0.0	0.0	6.0	30.0	10.0	1.5	5.0	90.12	88.10
18	1.0	0.0	0.0	0.0	0.0	10.0	30.0	10.0	1.5	5.0	48.56	43.24
19	0.0	−1.0	0.0	0.0	0.0	8.0	20.0	10.0	1.5	5.0	26.32	22.30
20	0.0	1.0	0.0	0.0	0.0	8.0	40.0	10.0	1.5	5.0	92.32	94.01
21	0.0	0.0	−1.0	0.0	0.0	8.0	30.0	5.0	1.5	5.0	75.20	73.57
22	0.0	0.0	1.0	0.0	0.0	8.0	30.0	15.0	1.5	5.0	127.88	126.17
23	0.0	0.0	0.0	−1.0	0.0	8.0	30.0	10.0	0.5	5.0	79.40	84.84
24	0.0	0.0	0.0	1.0	0.0	8.0	30.0	10.0	2.5	5.0	56.08	51.30
25	0.0	0.0	0.0	0.0	−1.0	8.0	30.0	10.0	1.5	2.0	14.56	16.88
26	0.0	0.0	0.0	0.0	1.0	8.0	30.0	10.0	1.5	8.0	128.04	124.16
27	0.0	0.0	0.0	0.0	0.0	8.0	30.0	10.0	1.5	5.0	53.16	53.49
28	0.0	0.0	0.0	0.0	0.0	8.0	30.0	10.0	1.5	5.0	53.24	53.49
29	0.0	0.0	0.0	0.0	0.0	8.0	30.0	10.0	1.5	5.0	52.80	53.49
30	0.0	0.0	0.0	0.0	0.0	8.0	30.0	10.0	1.5	5.0	52.94	53.49
31	0.0	0.0	0.0	0.0	0.0	8.0	30.0	10.0	1.5	5.0	50.86	53.49

The five-significant variables can be approximated by the quadratic model equation as follows:

(1) $$\begin{array}{l} \text{Y}=b_{0}+b_{1}{\text{X}}_{\text{1}}+b_{2}{\text{X}}_{2}+b_{3}{\text{X}}_{3}+{\text{b}}_{4}{\text{X}}_{4}+{\text{b}}_{5}{\text{X}}_{5}+{\text{b}}_{11}{\text{X}}_{1}^{2}\\ \;+{\text{b}}_{22}{\text{X}}_{2}^{2}+{\text{b}}_{33}{\text{X}}_{3}^{2}+{\text{b}}_{44}{\text{X}}_{4}^{2}+{\text{b}}_{55}{\text{X}}_{5}^{2}+{\text{b}}_{12}{\text{X}}_{1}{\text{X}}_{2}+{\text{b}}_{13}{\text{X}}_{1}{\text{X}}_{3}\\ \;+{\text{b}}_{23}{\text{X}}_{2}{\text{X}}_{3}+{\text{b}}_{14}{\text{X}}_{1}{\text{X}}_{4}+{\text{b}}_{24}{\text{X}}_{2}{\text{X}}_{4}+{\text{b}}_{34}{\text{X}}_{3}{\text{X}}_{4}+{\text{b}}_{15}{\text{X}}_{1}{\text{X}}_{5}\\ \;+{\text{b}}_{25}{\text{X}}_{2}{\text{X}}_{5}+{\text{b}}_{35}{\text{X}}_{3}{\text{X}}_{5}+{\text{b}}_{45}{\text{X}}_{4}{\text{X}}_{5}. \end{array}$$

Where Y are the response (BS production and oil displacement activity (ODA), respectively); Xj: system variables (correspond to the different factors influencing the production of BS) and *b*_0_, *b*_*j*_, *b*_*jk*_, and *b*_*jj*_: model coefficients.

Validation of the optimum BS production predicted by the CCD model was conducted in triplicate. The generation and the data treatment of the five factors CCD were performed using NemrodW software (Mathieu et al., 2000).

### BS purification, properties, and structural characterization

The supernatant of optimized fermentation culture was collected after centrifugation (12,000 rpm/20 min at 4°C), and was adjusted to pH 2.0 with 6N HCl. Then, the acidified supernatant was left overnight at 4°C in order to precipitate the BS. The precipitate was collected by centrifugation at 12,000 rpm for 30 min at 4°C to obtain the crude BS. For additional purification, the crude BS was extracted at three successive washes with a mixture of the chloroform-methanol solvent (2:1, v/v). Finally, the combined extracts were then dried with anhydrous sodium sulfate and were concentrated using a rotary evaporation at 40°C. The resulting product was obtained as a viscous brown matter. A portion of the viscous brown extract was then purified by silica gel column chromatography using sequential washes of chloroform and methanol (Smyth et al., 2010). The active fraction (5 ml each) was confirmed through two different tests [emulsification activity (E_24_) and ODA].

For emulsifying activity essays, a volume of 2 ml of vegetable oil was vigorously votexed with 2 ml of BS fraction in a screw-top glass tube for 2 min and allowed to stand for 24 h, and then the emulsification index (E_24_%) was calculated (Cooper and Goldenberg, 1987). A BS-producing strain having high emulsion stability (E_24_ ≥ 50%) was considered as an efficient emulsifying strain. The oil displacement activity was determined according to Techaoei et al. (2011), a volume of 20 μl of crude oil was placed on the surface of 20 ml of distilled water into the culture dish of diameter (9 cm) and a 10 μl of each fraction was gently placed on the surface of the oil film. Diameter of the clear halo viewed on oil surface under visible light was measured.

Rhamnolipid BS detection was performed by thin-layer chromatography TLC on pre-coated silica gel of standard 20 × 20 Kiesel-gel 60 F254 Merck plates using chloroform: methanol: acetic acid (65:15:2, v/v/v) as a solvent system and antrone as a visualization agent (Antoniou et al., 2015).

To confirm the glycolipid nature of the BS produced, Fourier Transform Infra-Red spectroscopy FTIR (Perkin Elmer FTIR model 2000) was applied as described by Rahman et al. (2010). This technique makes it possible to explore the functional groups and the chemical bonds present in the purified extract of BS. Infrared absorption spectra were obtained over the range of 400–4,000 cm^−1^ with a resolution of 4 cm^1^.

The stability studies of BS produced by the selected strain were carried out with respect to temperature, pH and salinity (Sharma et al., 2015). To determine the thermal stability of BS, the produced glycolipid BS was maintained at different temperature range of 20–90°C at neutral pH for 24 h; then the activity was measured. To evaluate the effect of pH on BS activity, BS solutions with different pH values (ranging from 2 to 10) were prepared using 100 mM buffers (tris-HCl pH 2–3, sodium acetate pH 4–5, phosphate pH 6–8 and tris-NaOH pH 9–10). Subsequently, the mixtures were vortexed and the ODA was measured after 24 h at room temperature. The effect of addition of different concentrations of salt (ranging from 0 to 600 mM NaCl) on the activity of produced BS was investigated at pH 7 and at room temperature.

### Enhancement of pesticide solubilization using S211 BS

The enhanced solubilization of Pentachlorophenol (PCP) into aqueous phase was evaluated using different BS concentrations (between 5 and 20 g/L) in comparison with three control preparations: a sample without BS and a sample with various concentrations of the synthetic surfactants, sodium dodecyl sulfate (SDS) and Tween 80 (between 5 and 20 g/L). For each experiment, in a 20 ml screw-capped glass vial, 0.5 g/L of PCP was suspended in deionized water (to a final volume of 10 ml). The vials were covered with aluminum foil to protect the surfactant samples from photolysis and were vigorously vortexed at room temperature (30°C). After 24 h, the residual pesticide remaining in the bottom of the tube was separated by centrifugation (15 min/12,000 rpm) and the aqueous phase was then transferred to clean vials until analysis. The PCP concentration was carried out using a reverse phase Ultra-High Performance Liquid Chromatography (UHPLC) equipped with a UV detector (at 265 nm). The separation was performed at 27°C on C18 column (250 × 4.6 mm; Inertsil ODS-4, GL Sciences, Japan) using an isocratic method with acetonitrile-water mixture of 50: 50 (v/v) as the mobile phase at a flow rate of 1 ml/min. The volume of the sample injected was 10 μl. The retention time was about 3.1 min. The enhanced apparent solubility of PCP by surfactant addition is expressed by an enhanced solubility ratio (ESR) (Wattanaphon et al., 2008), calculated according to the following formula:

(2) $$\text{ESR}(\text{PCP})={\text{C/C}}_{0}$$

Where C: the pesticide concentration in surfactant based solution and C_0_: the pesticide concentration in surfactant free solution.

### Statistical analysis

The statistical software package, NemrodW was performed to conduct a regression analysis of the obtained experimental data and to plot the response surface graphs (Mathieu et al., 2000). Student's *t*-test permitted to evaluate the statistical significance of the CCD model coefficients. Analysis of variance (ANOVA) using Fisher's *F*-test was performed on experimental data to check the statistical significance of the model.

### Nucleotide sequence accession number

This Whole Genome Shotgun project of *P. rhizophila* S211 has been deposited at DDBJ/ENA/GenBank under the accession CP024081.

## Results

### Isolation, phylogenetic assignment, general feature, and genomic insights inferred from S211 genome sequence

S211 is a free-living rhizobacterium isolated from a pesticide-contaminated soil through consecutive exposure to elevated concentrations of dimethoate and PCP in minimal salt medium. *In vitro* PGP experiments showed that S211 strain was able to grow under high salinity (10% NaCl) and alkalinity (pH 9), fix nitrogen, solubilize inorganic phosphate, synthetize auxine, HCN, ammonia, pyoverdine-siderophores and produce exopolysaccharides and anionic BS (Supplementary Figure 1).

**Figure 1:**
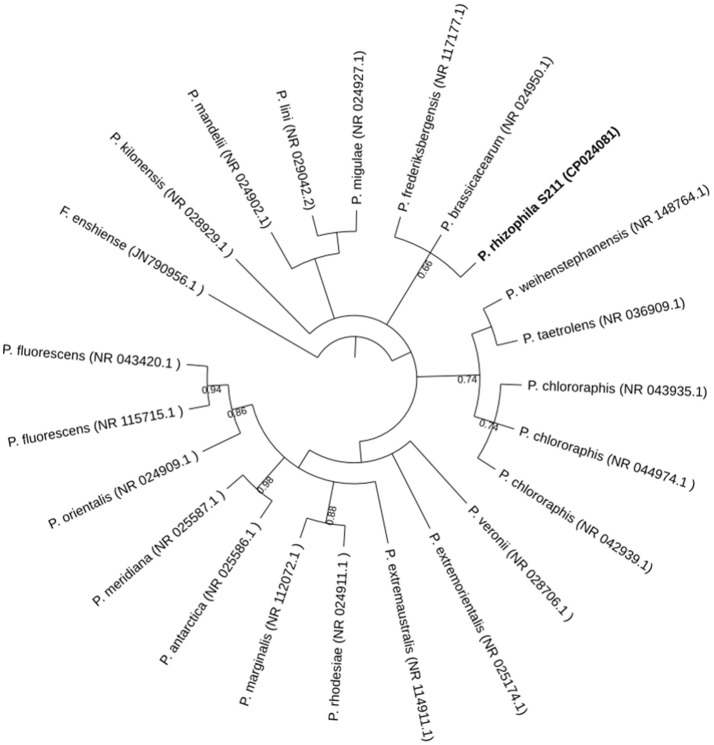
Phylogenetic tree generated with Mr. Bayes based on 16S rRNA gene sequences of *P. rhizophila* S211 isolate and related species. The tree was rooted with *Flavobacterium enshiense* (N790956.1) as an out-group. Bootstrap values are expressed as randomization of 1,000. GenBank accession numbers of the reference strains are indicated.

The phylogenetic relatedness to the closest relative species, based on almost-complete 16S rDNA gene sequences, showed that S211 is most closely to *P. frederiksbergensis* (NR_117177.1) and *P. brassicacearum* (NR_024950.1) with 99% sequence similarities for both (Figure 1). This value is higher than the 98.7% identity threshold to propose a new species. However, such comparison has the disadvantage that 16S rRNA sequence similarities do not always accurately reflect similarities at the whole-genome level and cannot distinguish between recently diverged species (Richter and Rosselló-Móra, 2009; Zhang and Qiu, 2016). Two methods, isDDH and ANI values, known for species delineation were used to confirm the phylogentic assignement. S211 strain genome is close to those of *P. brassicacearum* (CP012680.1) and *P. frederiksbergensis* (NZ_CP017886.1), with isDDH values of 35.50 and 26.20, and ANI values of 87.96 and 81.14%, respectively. Digital DDH values, lower than the threshold of 70% for species delineation, together with the ANI values, lower than the cut-off value of 95% distinguishing different species (Meier-Kolthoff et al., 2013a,b), indicate that *P. rhizophila* S211 forms a separate novel species within the genus *Pseudomonas*.

**Figure 2:**
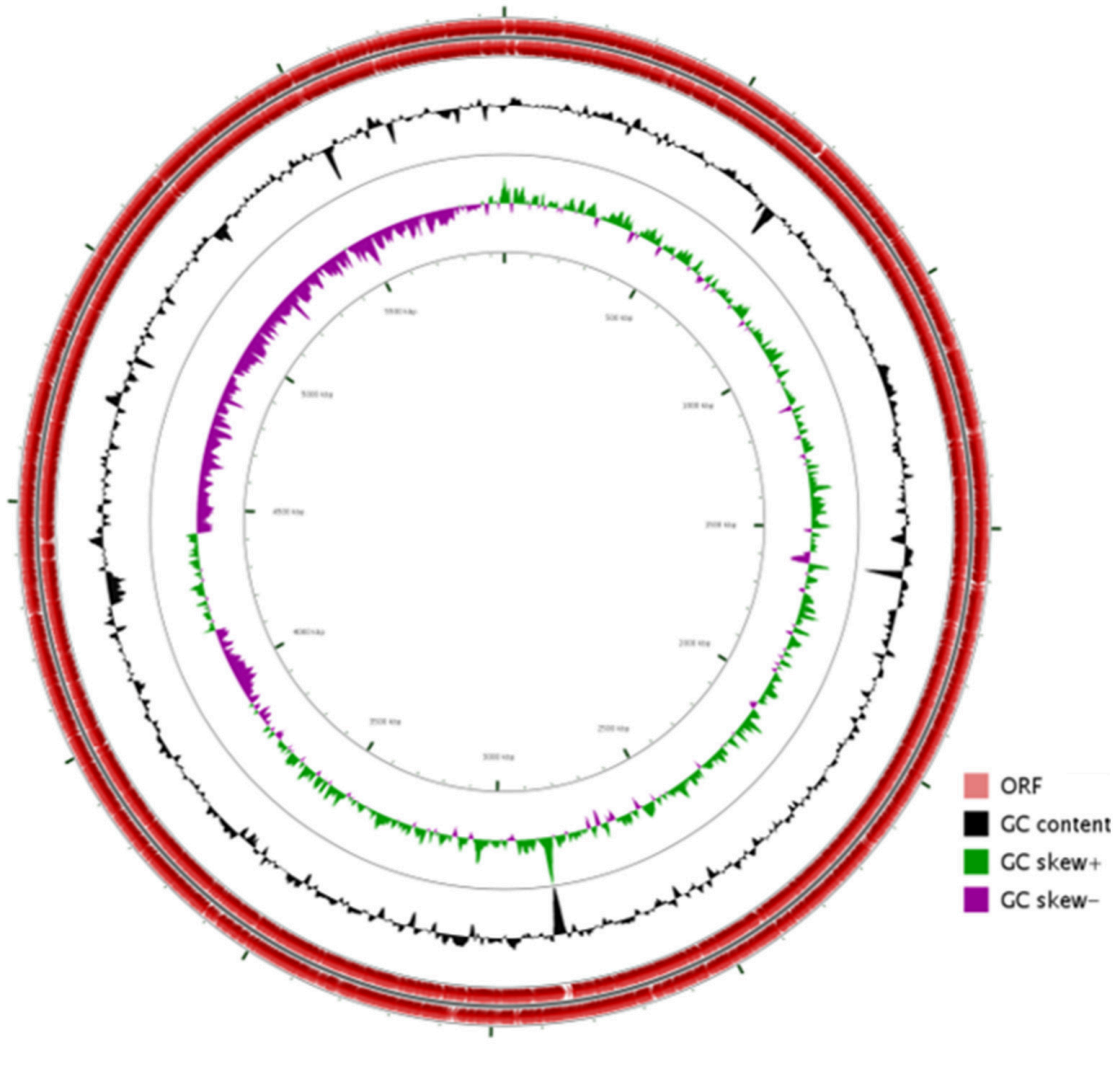
Circular representation of *P. rhizophila* S211 genome generated by CG viewer. Circles from outside to inside: first, scale bar in kilobases; second and third, predicted coding sequences of chromosome on leading and lagging strands; fourth, GC content; fifth, GC skew.

**Figure 3:**
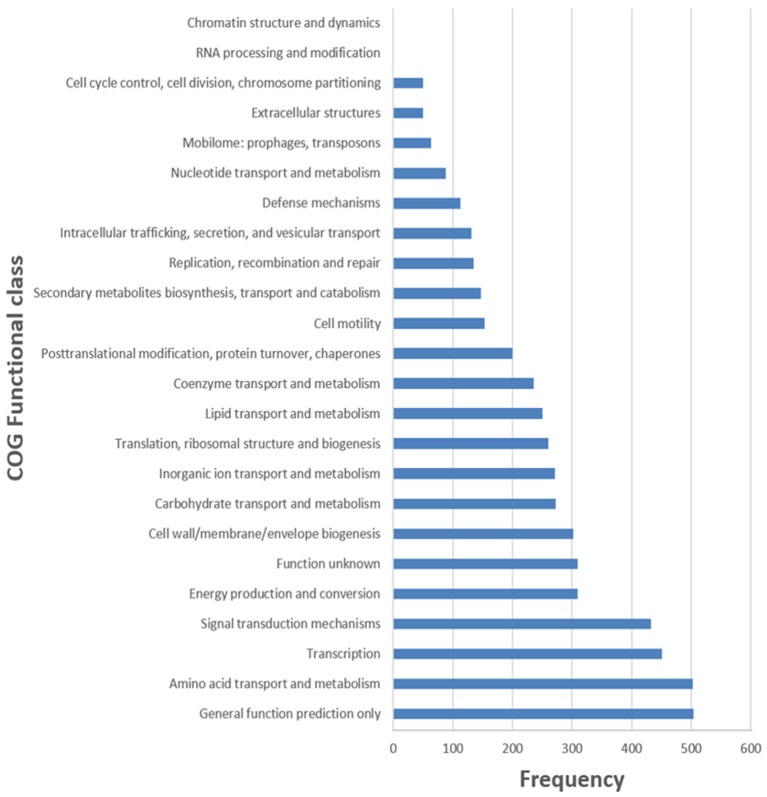
Frequencies of functional COG classes.

The general features of S211 genome sequence are shown in Table 2 and Supplementary Table 1. The genome consists of one scaffold with 5,948,515 bp (Figure 2) with an average GC content of 60.4%. Among the 5306 predicted genes, 4441 were identified as protein coding genes where 865 (15.67%) without function prediction. The CDSs were classified into 24 functional categories according to the COG database (Figure 3). Beside the predicted genes, a total of 57 tRNA, 9 rRNA loci (5S, 16S, 23S), and 547 SEED subsystem features were predicted in S211 genome sequence.

**Table 2:** Genome properties and features of S211 strain.

	**Total**	**Number**
DNA, total number of bases	5,948,515	100.00%
DNA coding number of bases	5,285,067	88.85%
DNA G+C number of bases	3,594,697	60.43%
DNA scaffolds	1	100.00%
Genes total number	5,521	100.00%
Protein coding genes	5,306	96.11%
RNA genes	215	3.89%
rRNA genes	9	0.16%
5S rRNA	6	0.11%
16S rRNA	2	0.04%
23S rRNA	1	0.02%
tRNA genes	57	1.03%
Other RNA genes	149	2.70%
Protein coding genes with function prediction	4,441	80.44%
Protein coding genes without function prediction	865	15.67%
Protein coding genes with enzymes	1,371	24.83%
Protein coding genes w/o enzymes but with candidate KO based enzymes	5	0.09%
Protein coding genes connected to KEGG pathways	1,610	29.16%
Protein coding genes not connected to KEGG pathways	3,696	66.94%
Protein coding genes connected to KEGG Orthology (KO)	2,927	53.02%
Protein coding genes not connected to KEGG Orthology (KO)	2,379	43.09%
Protein coding genes connected to MetaCyc pathways	1,162	21.05%
Protein coding genes not connected to MetaCyc pathways	4,144	75.06%
Protein coding genes with COGs	3,954	71.62%
Protein coding genes with KOGs	1,031	18.67%
Protein coding genes with Pfam	4,613	83.55%
Protein coding genes with TIGRfam	1,762	31.91%
Protein coding genes with InterPro	2,959	53.60%
Protein coding genes with IMG Terms	1,467	26.57%
Protein coding genes with IMG Pathways	455	8.24%
Protein coding genes with IMG Parts List	599	10.85%
Protein coding genes in internal clusters	1,497	27.11%
Protein coding genes in Chromosomal Cassette	5,457	98.84%
Chromosomal Cassettes	444	–
Biosynthetic Clusters	8	–
Genes in Biosynthetic Clusters	205	3.71%
Fused Protein coding genes	724	13.11%
Protein coding genes coding signal peptides	559	10.12%
Protein coding genes coding transmembrane proteins	1,224	22.17%
COG clusters	2,067	52.28%
KOG clusters	562	14.21%
Pfam clusters	2,603	56.43%
TIGRfam clusters	1,390	78.89%

**Figure 4:**
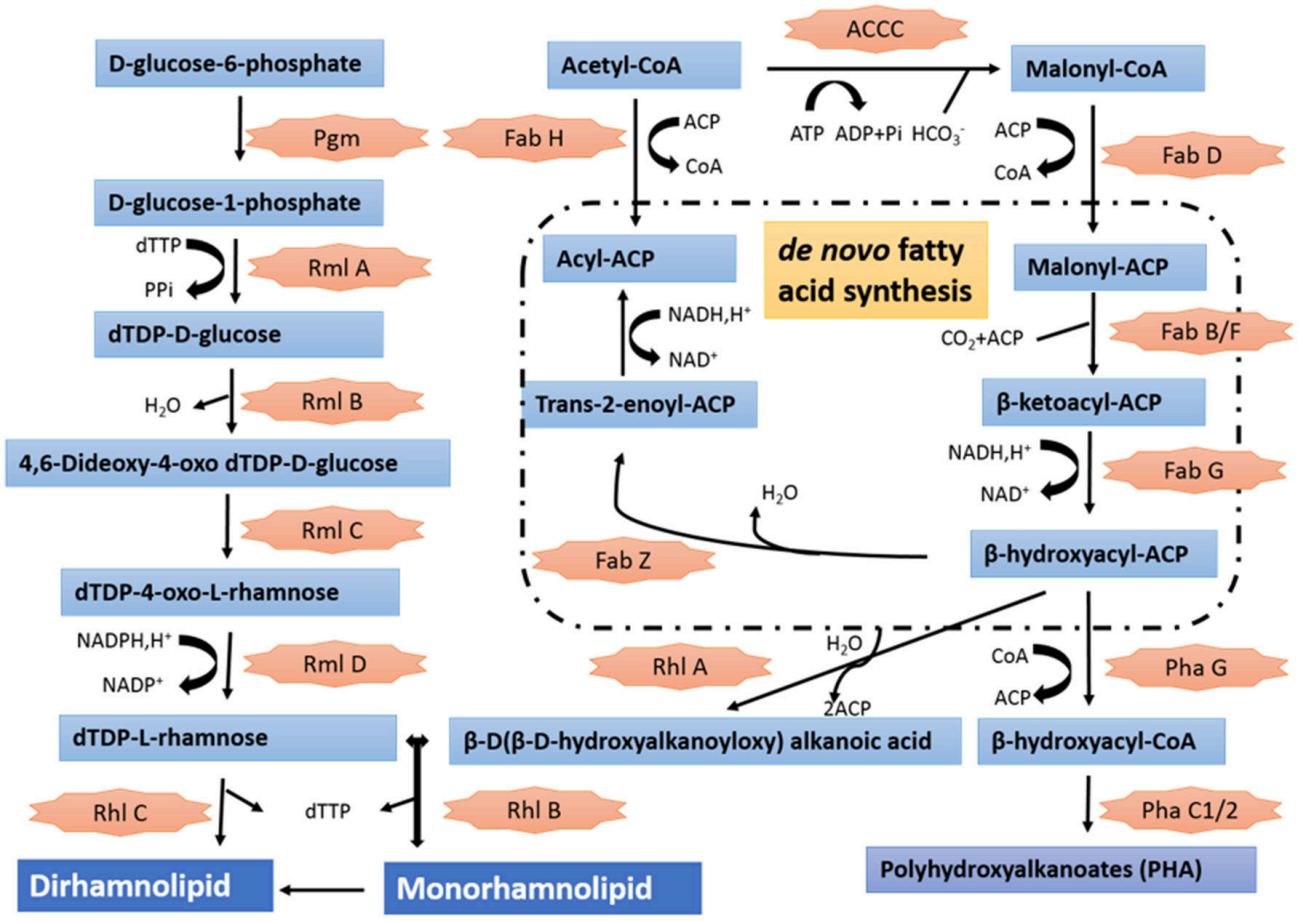
Putative metabolic pathway involved in the synthesis of rhamnolipid in genome of *P. rhisophila* S211. Pgm, Phosphoglucomutase, EC 5.4.2.2; RmlA, Glucose-1-phosphate thymidylyltransferase, EC 2.7.7.24; RmlB, dTDP-glucose 4,6-dehydratase, EC 4.2.1.46; RmlC, dTDP-4-dehydrorhamnose 3,5-epimerase, EC 5.1.3.13; RmlD, dTDP-4-dehydrorhamnose reductase, EC 1.1.1.133; RhlB and RhlC, Glycosyltransferase, EC 2.4.1.; PhaG, (R)-3-hydroxydecanoyl-ACP:CoA transacylase PhaG (3-hydroxyacyl-CoA-acyl carrier protein transferase), EC 2.4.1.-; PhaC1/2: Polyhydroxyalkanoic acid synthases, EC 2.3.1-; ACCC, Acetyl-coenzyme A carboxyl transferase alpha chain, EC 6.4.1.2; FabD, Malonyl CoA-acyl carrier protein transacylase, EC 2.3.1.39; Fab H, 3-oxoacyl-[acyl-carrier-protein] synthase, KASIII, EC 2.3.1.180; FabB/F, 3-oxoacyl-[ACP] synthase, EC 2.3.1.41; FabG, 3-oxoacyl-[acyl-carrier protein] reductase, EC 1.1.1.100.

Annotation of the draft genome sequence confirmed the presence of genes involved in plant-growth promoting, biological control and bioremediation activities (Supplementary Table 2). S211 genome contained several genes specific to nitrogen fixation and others encoding alkaline phosphatases, which convert insoluble phosphorus into a bioavailable form for plant growth. The genome of S211 also contained putative 1-aminocyclopropane-1-carboxylate (ACC) deaminase. Different kinds of defenses are revealed in the genome of the PGPR strain S211, including genes involved in resistance/tolerance to antibiotics, heavy metals and toxic compounds (90), oxidative stress (99), osmotic stress (34), temperature stress (23), and multiple drugs (28). *P. rhizophila* S211 can produce bacteriocins, ribosomally synthesized antibacterial peptides as well as several antagonistic molecules such as phenazines. S211 can also synthesize pyroverdine, a fluorescent siderophore, and chelate the ferric iron Fe(III) under low-iron conditions and thereby make it available for microbial and plant cells. The genome of S211 harbors 18 genes encoding dioxygenases (DOs) related to degradation of aromatic compounds. *P. rhizophila* is able also to produce and excrete the exopolysaccharide levan mediated by extracellular levan sucrase. The rhamnolipid synthesis proceeds by sequential glycosyl transfer reactions, catalyzed by glycosyl transferases with TDP-rhamnose acting as a rhamnosyl donor and 3-hydroxydecanoyl-3-hydroxydecanoate acting as the acceptor. All enzymatic steps required for the synthesis of rhamnolipids by *P. rhizophila* S211 are available in Figure 4.

### Optimization of BS production by *P. rhizophila* S211 on OMWW based medium using response surface methodology

In this investigation, the power of response surface method to optimize BS production by *P. rhizophila* S211 was explored using a five variables CCD (Table 1). The experimental results were modeled with a second-order polynomial equation to predict the response for given experimental conditions within the design space:

(3) $$\begin{array}{l} \text{BS yield}(\text{mg/L}):\text{Y}=53.493-22.431{\text{X}}_{1}+35.853{\text{X}}_{2}\\ \;+26.302{\text{X}}_{3}-16.769{\text{X}}_{4}+70.524{\text{X}}_{5}\\ \;+12.185{\text{X}}_{1}^{2}+4.665{\text{X}}_{2}^{2}\\ \;+46.385{\text{X}}_{3}^{2}+14.585{\text{X}}_{4}^{2}+0.145{\text{X}}_{5}^{2}\\ \;-54.250{\text{X}}_{1}{\text{X}}_{2}-58.820{\text{X}}_{1}{\text{X}}_{3}\\ \;+44.140{\text{X}}_{2}{\text{X}}_{3}+37.120{\text{X}}_{1}{\text{X}}_{4}\\ \;-59.020{\text{X}}_{2}{\text{X}}_{4}-49.730{\text{X}}_{3}{\text{X}}_{4}\\ \;-45.760{\text{X}}_{1}{\text{X}}_{5}+30.780{\text{X}}_{2}{\text{X}}_{5}\\ \;+23.670{\text{X}}_{3}{\text{X}}_{5}-14.850{\text{X}}_{4}{\text{X}}_{5}. \end{array}$$

where Y was the estimated BS production and X_1_, X_2_, X_3_, X_4_, and X_5_ were the coded values for pH, temperature, OMWW concentration, inoculum size, and incubation time, respectively.

Statistical analysis of experimental results was performed with NomrodW statistical software to determine the significant differences between the independent variables. The significance of CCD model coefficients was determined by *t*-values and *p*-values which indicates the pattern of interactions between the five variables. The Student *t* distribution and the corresponding *p*-values, along with the variable estimate, were illustrated in Table 3. In this case, the linear effect of the parameters X_2_ and X_5_ and the interactions X_1_X_2_, X_1_X_3_, X_1_X_4_, X_1_X_5_, X_2_X_3_, X_2_X_4_, and X_3_X_4_ were statistically significant. By considering only these significant factors, BS production by *P. rhizophila* S211 can be predicted by the following equation:

(4) $$\begin{array}{l} \text{Y}=53.493+35.853{\text{X}}_{2}+70.524{\text{X}}_{5}-54.250{\text{X}}_{1}{\text{X}}_{2}\\ \;-58.820{\text{X}}_{1}{\text{X}}_{3}+44.140{\text{X}}_{2}{\text{X}}_{3}+37.120{\text{X}}_{1}{\text{X}}_{4}\\ \;-59.020{\text{X}}_{2}{\text{X}}_{4}-49.730{\text{X}}_{3}{\text{X}}_{4}-45.760{\text{X}}_{1}{\text{X}}_{5}. \end{array}$$

The significance of the CCD model was checked by *F*-test and the corresponding statistical results were presented in Supplementary Table 3. ANOVA analysis for BS production showed that the regression model was significant and the lack of fit was insignificant (Supplementary Table 3). The fit of the models was evaluated by the determination of coefficient R^2^. The regression equations obtained indicated the R^2^ values of 0.938 suggesting an adequate adjustment of the quadratic model to the experimental data and indicating that the model could explain 93.80% of the variability in the response.

**Table 3:** Estimated effect, regression coefficient, and corresponding *t*- and *P*-values for BS production in central composite design experiments.

**Name**	**Coefficient**	**F. Inflation**	**Stand. Dev**.	**t.exp**.	**Signification %**
b0	53.493		16.994	3.15	^*^
b1	−22.431	1.00	13.431	−1.67	12.3%
b2	35.853	1.00	13.431	2.67	^*^
b3	26.302	1.00	13.431	1.96	7.6%
b4	−16.769	1.00	13.431	−1.25	23.9%
b5	70.524	1.00	13.431	5.25	^***^
b11	12.185	3.07	36.341	0.34	74.2%
b22	4.665	3.07	36.341	0.13	89.6%
b33	46.385	3.07	36.341	1.28	22.9%
b44	14.585	3.07	36.341	0.40	69.7%
b55	0.145	3.07	36.341	0.00	99.2%
b12	−54.250	1.00	14.246	−3.81	^**^
b13	−58.820	1.00	14.246	−4.13	^**^
b23	44.140	1.00	14.246	3.10	^*^
b14	37.120	1.00	14.246	2.61	^*^
b24	−59.020	1.00	14.246	−4.14	^**^
b34	−49.730	1.00	14.246	−3.49	^**^
b15	−45.760	1.00	14.246	−3.21	^**^
b25	30.780	1.00	14.246	2.16	5.4%
b35	23.670	1.00	14.246	1.66	12.5%
b45	−14.850	1.00	14.246	−1.04	32.3%

*** Significant at the level 99.9%;

** Significant at the level 99%;

* *Significant at the level 95%; NS, Non-Significant*.

**Figure 5:**
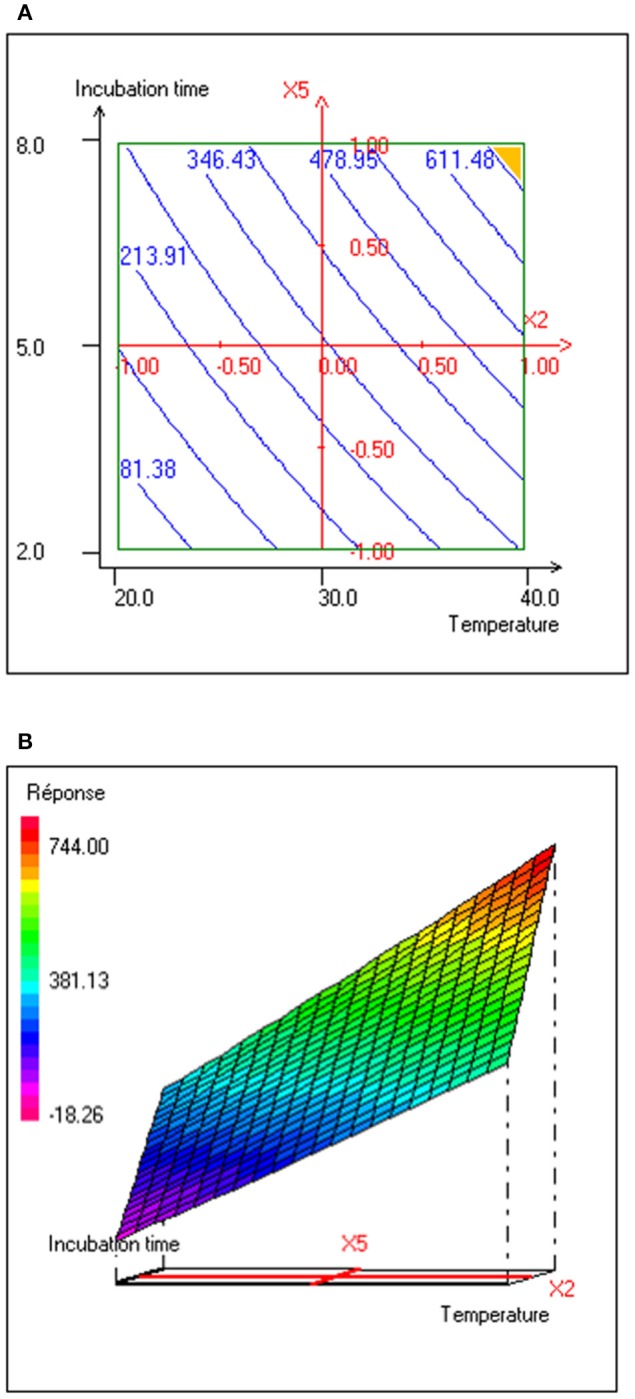
**(A)** Contour plot and **(B)** response surface plot of interaction effect between temperature (°C) and incubation time (days) on BS production yield (mg/L) with pH, OMWW concentration and inoculum size kept at 6.0, 15% and 0.5%, respectively.

**Figure 6:**
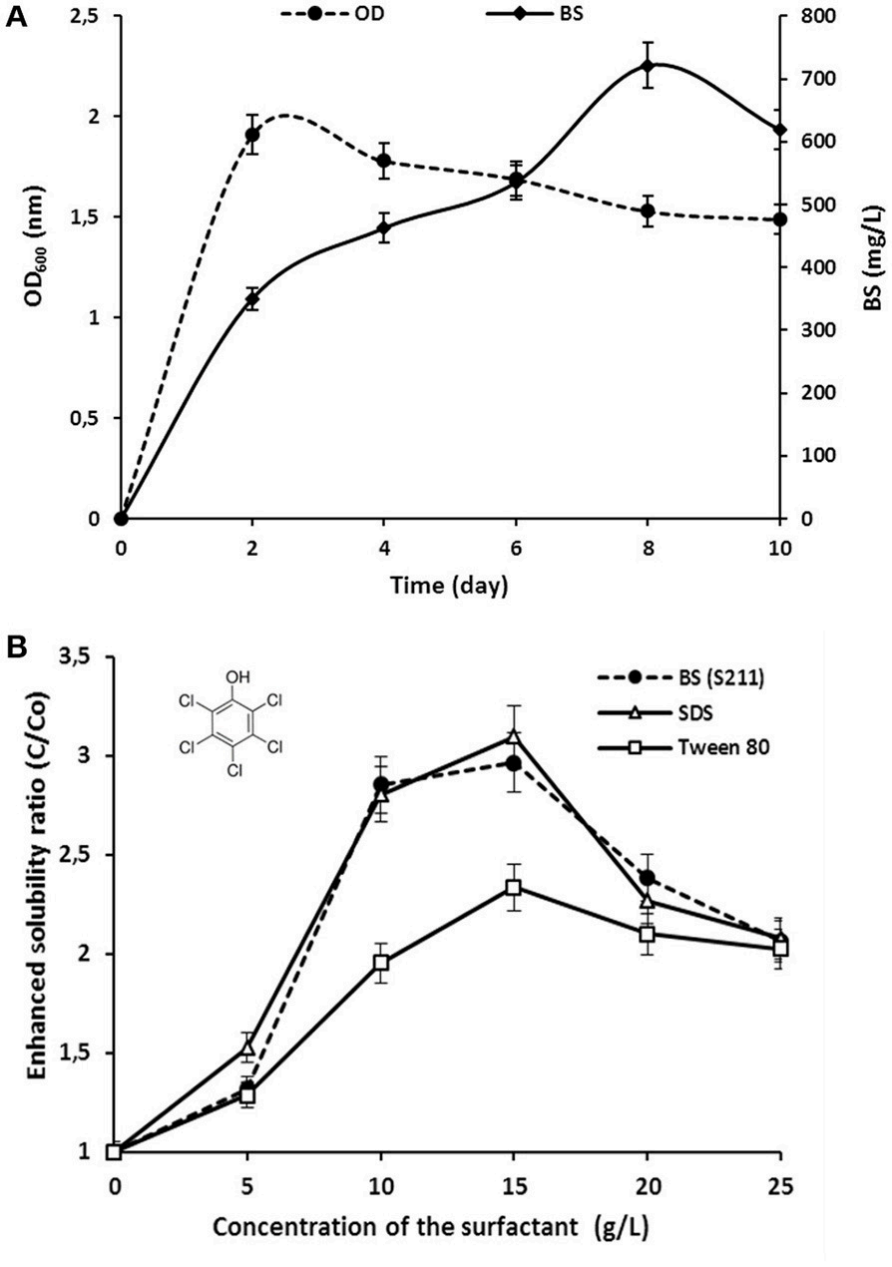
**(A)** Growth (OD_600nm_) kinetics and BS production by *P. rhizophila* S211 under optimized conditions; **(B)** Effect of chemical surfactants and *P. rhizophila* rhamnolipid BS concentrations on the solubility of PCP.

The interaction effects and optimal levels of the selected culture variables on BS production by *P. rhizophila* S211 were determined by plotting the response surface curves against the two significant variables X_2_ and X_5_, while fixing the other variables at constant levels (pH 6, OMWW 15% and inoculum size 0.5%). The contour plots and response surface curves for the predicted response Y (BS production yield), based on the second-order model were shown in Figure 5. They provided useful information about interactions between variables and allowed an easy interpretation of the CCD results and prediction of the optimal levels of each variable for maximum BS production (the highlighted zone in yellow). Indeed, S211 BS yield increased with the increase in temperature and incubation time from their low levels to their high levels (Figure 5). As a result, based on the response surface and contour plots, the optimum operating culture conditions, carried out numerically by using NemrodW software were: OMWW concentration 15%, inoculum size 0.5%, temperature 40°C, pH 6.0 and incubation time 8 days. The expected value of the BS production yield was Y1 = 721.47 ± 43 mg/L. A supplementary experiment was performed under the selected optimal production conditions. It led to BS production yield equal to 720.80 ± 55.90 mg/L, which was well in close agreement with predicted value (Figure 6A).

### Biochemical characterization of purified BS from *P. rhizophila* S211 and its application in enhancement of pesticide solubilization

The purified BS of *P. rhizophila* S211 showed important emulsification activity (E24 = 90%) and oil displacement area (ODA = 63.58 cm^2^). The separation of purified BS products on TLC plate indicate the presence of two characteristic spots, when anthrone reagent was sprayed. According to the commercially available purified rhamnolipid, the lower spot consisted of di-rhamnolipids with a retention factor value equal to 0.43, while the higher spot consisted of mono-rhamnolipids with a retention factor value equal to 0.67.

**Figure 7:**
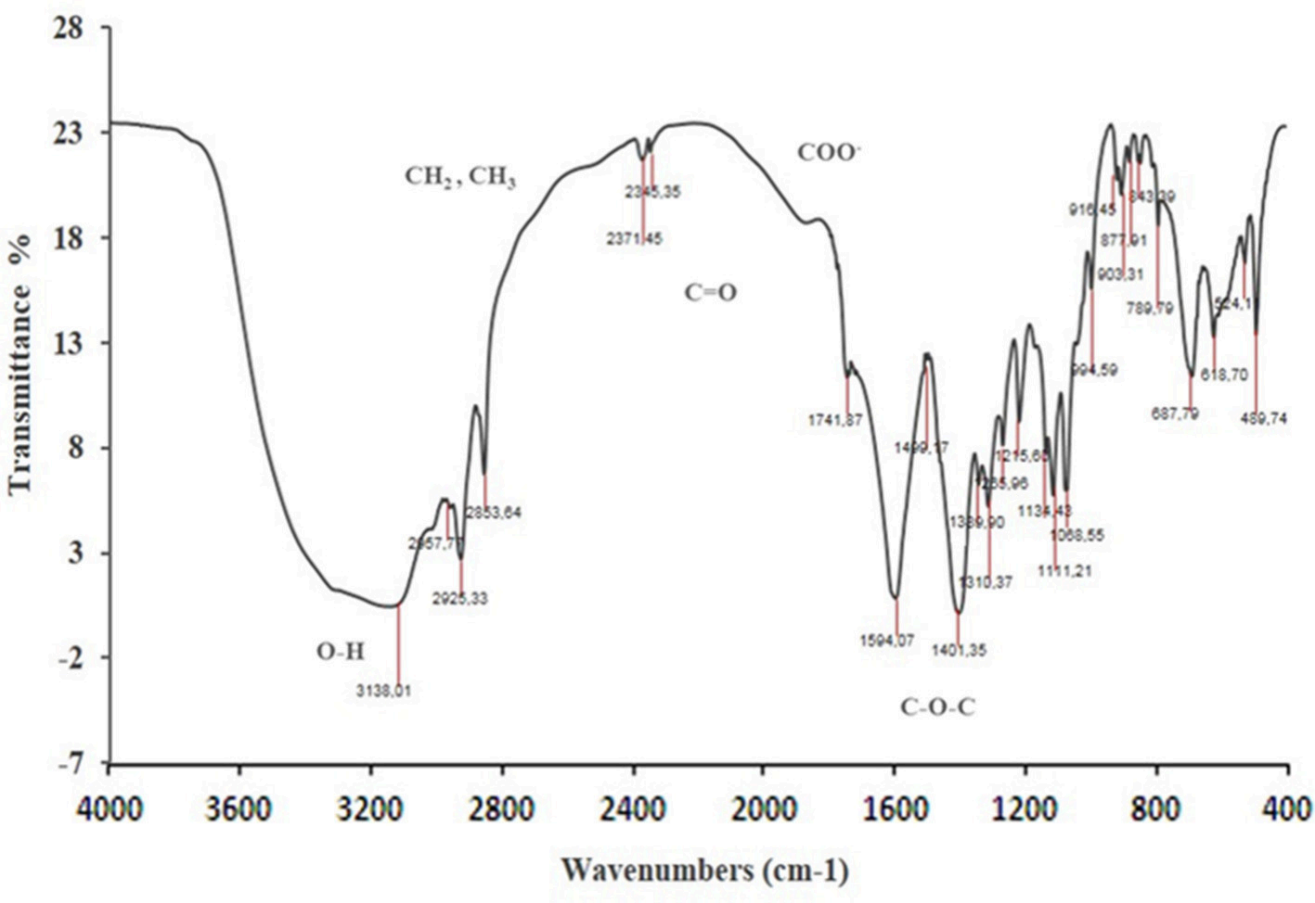
Fourier Transform InfraRed spectroscopy analysis of BS produced by *P. rhizophila* S211.

FTIR characteristic peaks of *P. rhizophila* BS observed at 3138 denoted the presence of –OH stretching (free hydroxyl groups of rhamnose rings) of hydroxyl group. The strong adsorption peaks at 2345, 2925, and 2853 showed the presence of methylene and the terminal methyl group of aliphatic (-CH, -CH_2_, -CH_3_) stretching bands confirmed the glycolipid type of produced BS (Figure 7). The carbonyl functional group (C = O) had a peak in the region of 1,741 cm^−1^. Peaks recorded in the range of 1,200–1,000 cm^−1^ indicate the presence of bonds between carbon atoms and the hydroxyl groups found in the rhamnose units.

**Figure 8:**
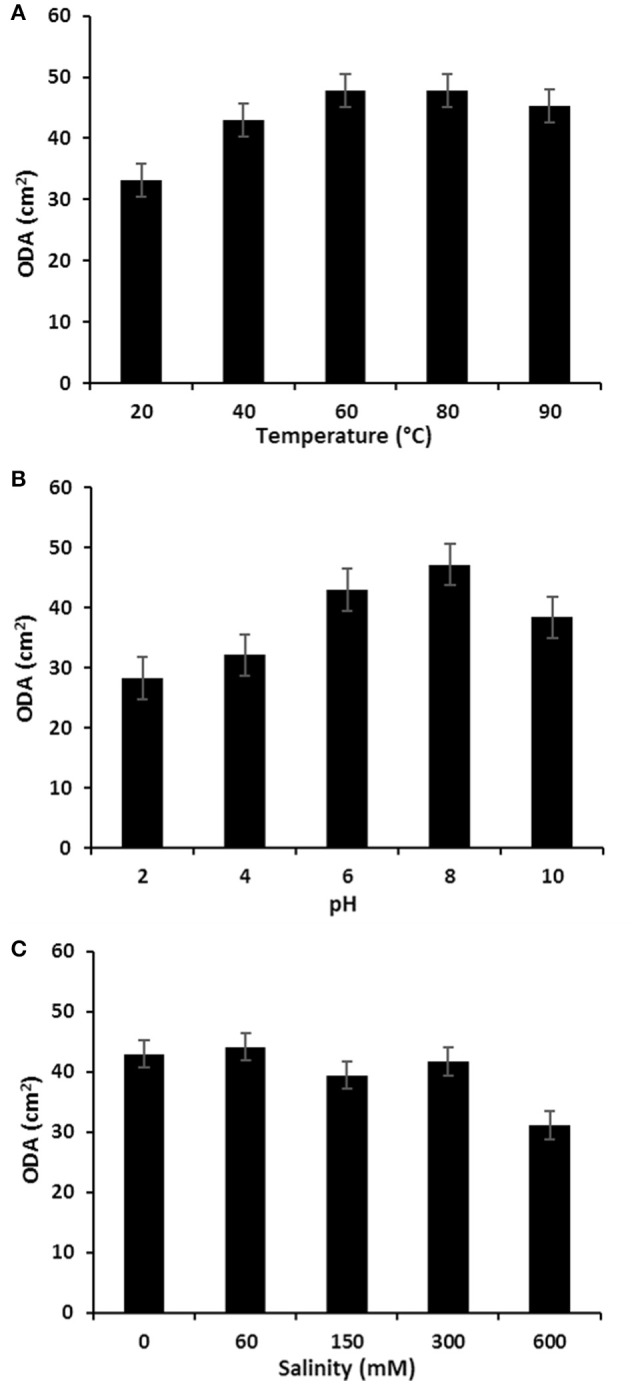
**(A)** Temperature; **(B)** pH, and **(C)** salinity effects on the stability of glycolipid BS produced by *P. rhizophila* S211.

The stability of *P. rhizophila* BS at different salinity, pH and temperature values was measured and the results are illustrated in Figure 8. The ODA of purified BS showed a remarkable increase at high temperatures and remained without any significant decrease on the oil displacement capacity in a range of 60–80°C, therefore it was found that the *P. rhizophila* BS is thermally stable. In addition, the salinity and pH stability analysis were carried out, revealing that *P. rhizophila* BS conserves its surfactant activity up to 300 mM NaCl. On the other hand, the purified BS produced by *P. rhizophila* S211 showed higher stability at alkaline conditions than acidic conditions.

The ability of purified BS from *P. rhizophila* S211 to enhance PCP solubility was performed in comparison with those of synthetic surfactants, i.e., a nonionic surfactant (Tween 80) and an anionic surfactant (SDS) (Figure 6B). The aqueous solubility of PCP was evaluated by test tube solubilization assays in the presence of different concentrations of surfactants ranging from 5 to 25 g/L. For all surfactants tested, the apparent PCP solubility increased with an increase in BS concentration up to 15 g/L. With an initial PCP concentration of 500 mg/L, enhanced PCP solubility ratios were about 2.32 ± 0.01, 3.15 ± 0.03, and 2.96 ± 0.02 folds higher than the pure water solubility for Tween 80, SDS and S211 BS, respectively. SDS was more effective than Tween 80 in enhancing PCP solubilization. Under these conditions, BS from *P. rhizophila* S211 showed better solubilization efficiency than Tween 80, while exhibited fairly similar behavior to an anionic surfactant, SDS, which confirm the anionic nature of BS from S211 strain.

## Discussion

Although PGPR are mainly considered for promoting the plant growth and disease control, much attention has recently been focused on xenobiotic bioremediation using PGPR (Bishnoi, 2015). In an effort to develop innovative technological and management strategies for wastewater treatment and efficient reuse in agriculture, the MADFORWATER project (http://www.madforwater.eu/fr/) has been established. In the framework of this project, an extensive collection of PGPR isolates with bioremediation potential has been conducted. Among the group of PGPR, the genus *Pseudomonas* is strongly represented in literature. For example, Berendsen et al. (2015) reported that over 300 publications in the past 30 years described biological mechanisms involved in the ability of three PGPR strains *P. putida* WCS358, *P. fluorescens* WCS374, and *P. fluorescens* WCS417 to enhance plant growth and protect plants against diseases. In the present work, a novel species of *Pseudomonas*, namely *P. rhizophila* S211 has been isolated from a pesticide-contaminated artichoke farm soil in Tunisia using enrichment culture technique. *P. rhizophila* S211 appeared to be taxonomically very closely related to other plant growth-promoting *Pseudomonas* strains such as *P. putida* (Berendsen et al., 2015), *P. fluorescens* (Alsohim et al., 2014), and *P. aeruginosa* (Bhakthavatchalu et al., 2013; Singh and Cameotra, 2013).

*In vitro* biochemical experiments and genomic analysis showed that the free-living rhizobacterium, *P. rhizophila* S211 has a strong capacity to enhance plant growth mainly by increasing nitrogen fixation, phosphate solubilization, ACC deaminase and phytohormones production. Such plant growth-promoting activities of native *Pseudomonas* strains isolated from rhizospheric soils have been previously reported by many researchers (Beneduzi et al., 2012; Santoro et al., 2015; Kaundal et al., 2017). The biological control activity of this strain was mainly linked to siderophore-mediated competition for iron (Berendsen et al., 2015; Santoro et al., 2015). Among most of siderophore-producing bacteria studied, pyoverdine-synthesizing pseudomonads are known for their high affinity to the ferric ion. Several studies reported that fluorescent pseudomonads can efficiently colonize roots and suppress soil-borne fungal pathogens through the release of iron-chelating pyoverdines (Beneduzi et al., 2012).

*P. rhizophila* S211 exhibited bioremediation potentialities by synthesizing dioxygenases (DOs) and producing rhamnolipid BSs. The DOs including extradiol dioxygenase, homogentisate 1,2-dioxygenase, protocatechuate 3,4-dioxygenase, and aromatic ring-cleaving dioxygenases. The DOs play key roles in modifying diverse recalcitrant aromatic compounds to common intermediates, that can feed into central pathways. The ability to catabolize various recalcitrant aromatic compounds such as chemical pesticides in exudates represents one possible strategy that could confer a selective advantage in the plant rhizosphere (Shen et al., 2013). Rhamnolipids may also enhance bioremediation of pesticides and other pollutants in the rhizosphere by increasing the substrate availability for microorganisms, or by enhancing the hydrophobicity of the cell surface, allowing hydrophobic substrates to associate more easily with bacterial cells (Pacwa-Płociniczak et al., 2014). Rhamnolipid biosynthesis pathway is divided into biosynthesis of the fatty acid; sugar moieties and link the sugar and lipid (Wittgens et al., 2011; Irorere et al., 2017). In genome of strain S2111, the lipid moiety of the BS was generated through the classical pathway of fatty acid synthesis. All the genes potentially involved in the biosynthesis of dTDP-L-rhamnose were found based on S211 genome analysis including *pgm, rmlA, rmlB, rmlC*, and *rmlD*. The genes encoding rhamnosyltransferases that participate in the final steps of rhamnolipid biosynthesis were also identified in S211 genome. Rhamnolipid BS producers belonging to *Pseudomonas* species have been previously reported for *P. putida* (Kaskatepe et al., 2017)*, P. aeruginosa* (Pansiripat et al., 2010), *P. stutzeri* (Joshi and Shekhawat, 2014), *P. luteala* (Onbasli and Aslim, 2009), *P. fluorescens* (Peter and Singh, 2014), *P. chlorophis* (Lan et al., 2015), *P. alcaligenes* (Oliveira et al., 2009). However, this is the first study to describe PGP potential and BS synthesis by *P. rhizophila*.

*Pseudomonas* strains are able to use different substrates, such as glucose, fructose, glycerol, mannitol, n-paraffins and vegetable oils, cheaper agro-industrial wastes and by-products to produce rhamnolipid-type BS (Ben Belgacem et al., 2015; Gudiña et al., 2015a,b). In this study, the BS production by S211 was performed in low-cost liquid medium formulated with OMWW. The effects of pH, temperature, OMWW concentration, inoculum size, and incubation time and their interactions on maximization of *P. rhizophila* BS production were evaluated and validated, experimentally using a CCD and RSM. Based on this experimental design, the BS yields under each set of conditions were determined and compared with the corresponding predicted levels suggested by NemrodW software. The obtained results showed that the model can be used for the navigation of BS model space. The optimum conditions for maximum BS yield by *P. rhizophila* S211 (720 mg/L) were predicted from the produced model as follows: 15% for OMWW concentration, 0.5% for inoculum size, 40°C for temperature, 6.0 for pH and8 days for incubation time. The maximum BS production by *P. rhizophila* S211 cultivated on OMWW as an alternative low-cost substrate was relatively higher than those of *P. fluorescens* strains cultivated on high-cost hydrophobic substrates such as soybean oil (437 mg/L), coconut oil (299 mg/L), palm oil (289 mg/L), mustard oil (233 mg/L), sunflower oil (187 mg/L), and olive oil (108 mg/L) (Peter and Singh, 2014). Other researchers reported BS production by different *P. aeruginosa* strains using culture media containing agro-industrial by-products and vegetable oils, such as olive oil, sunflower oil, Babassu oil, palm oil and soybean oil, leading to BS yields between 200 and 800 mg/L (Kaskatepe and Yildiz, 2016).

Purified BS of S211 was initially characterized by TLC revealing double spots when being visualized under UV light, which confirmed the presence of glycolipids. The replica plate when sprayed with anthrone, produced dark and light brown spots indicating the presence of mono- and di-rhamnolipids. The molecular composition of the purified S211 BS was evaluated by FTIR, which revealed the presence of saccharides and lipid in combination. Similar results have been reported for other glycolipid BS produced by *Pseudomonas* strains (Banat et al., 2000; Rahman et al., 2010; Wittgens et al., 2011; Ibrahim et al., 2013; Nalini and Parthasarathi, 2014; Elazzazy et al., 2015).

Applicability of glycolipid BS in several fields depends on their activity and stability at harsh conditions (Banat et al., 2000; Mata-Sandoval et al., 2002; Manivasagan et al., 2014). The BS produced by *P. rhizophila* S211 exhibited good stability over higher temperatures, a large range of pHs and salt concentrations, making it a potential candidate for bioremediation of soils contaminated by xenobiotics. It was shown to be highly thermostable. In fact, heating of the BS sample to 100°C (or its autoclaving at 120°C) caused little significant decrease on the BS performance (Rahman et al., 2010; Techaoei et al., 2011). Little changes were also observed with addition of up to 300 mM sodium chloride. pH increase has a positive effect on S211 BS performance. This is due to higher stability of fatty acids-surfactant micells in the presence of sodium hydroxide and the precipitation of secondary metabolites at high pH values. The effect of pH on BS activities of different *Pseudomonas* strains has been well reported (Manivasagan et al., 2014; Elazzazy et al., 2015).

This study demonstrated that BS produced by plant-growth promoting *P. rhizophila* could enhance the solubility of PCP under different contamination conditions indicating the feasibility of BS application as an efficient biological tool for remediation of pesticide-contaminated site. Similar results were reported by Wattanaphon et al. (2008) who evaluated the solubilization potential of three pesticides, i.e., methyl parathion, trifluralin and ethyl parathion, in the presence of three surfactants namely Tween 80, SDS and a glycolipid produced by *Burkholderia cenocepacia* BSP3.

## Conclusion

Given their ability to enhance soil nutrient availability, produce plant growth–stimulating compounds, and protect against pathogens, PGPR are widely used as bioinoculants to support survival and development of plants even under various stressing conditions, such as pesticide contamination of soil. In this work, the new bacterium *P. rhizophila* S211, isolated from an agricultural contaminated soil, both displayed pesticide solubilizing and plant-growth-promoting activities. By analyzing the complete S211 genome, we identified the key genes that potentially promote plant growth as well as genes involved in xenobiotic biodegradation and BS synthesis. BS production by the new producer *P. rhizophila* S211 was performed in low-cost liquid medium supplemented with OMWW and optimized by RSM. *P. rhizophila* BS not only exhibited excellent yield (720 mg/L) but also showed high emulsification index (90%) and oil displacement area (63.58 cm^2^). S211 BS characterization using TLC and FT-IR confirmed the presence of the rhamnolipid type BS. It exhibited also good stability over higher temperatures, different pHs and sodium chloride. This glucolipid BS from *P. rhizophila* S211 noticeably enhanced PCP solubilization suggesting its role in environmental pesticide bioremediation. Further comparative genomic analysis with phylogenetically closely related strains together with structural and functional studies will allow for a more comprehensive understanding of the mechanisms used by this bacterium to remediate rhizospheric pollutants and to promote plant growth especially for the crops grown in contaminated soils. A future research is also needed to elucidate glycolipid BS-pesticide interactions in soil to find out predictive and mechanistic models and thus better real-scale bioremediation design.

## Author contributions

Conceived and designed the experiments: MN, HbC, HnC, YS, NR, FF, and AC. Analyzed the data: WH, MN, AN, RC, AS, FN, AM, and NR. Contributed reagents, materials, analysis: WH, HnC, AS, YS, and NR. Manuscript preparation and revision: WH, MN, NR, HO, and AC.

### Conflict of interest statement

The authors declare that the research was conducted in the absence of any commercial or financial relationships that could be construed as a potential conflict of interest.
