# Supplementary material for: Pseudomonas rhizophila S211, a New Plant Growth-Promoting Rhizobacterium with Potential in Pesticide-Bioremediation
Source: Front Microbiol. 2018 Feb 23;9:34. doi: 10.3389/fmicb.2018.00034 (PMC5829100; doi:10.3389/fmicb.2018.00034)
Supplement: Supplementary file 1 [file Table1.DOC]

**Supplementary Table 1. Classification and general features of *P. rhizophila* strain S211.**

| **MIGS ID** | **Property** | **Term** |
| --- | --- | --- |
|  | Classification | Domain *Bacteria* |
|  |  | Phylum *Proteobacteria* |
|  |  | Class *Gammaproteobacteria* |
|  |  | Order *Pseudomonadales* |
|  |  | Family *Pseudomonadaceae* |
|  |  | Genus *Pseudomonas* |
|  |  | Strain: S211 |
|  | Gram stain | Negative |
|  | Cell shape | Rod |
|  | Motility | Motile |
|  | Sporulation | Not reported |
|  | Temperature range | 4 °C–50 °C |
|  | Optimum temperature | 30 °C |
|  | pH range; Optimum | 5.0–9.0; 7 |
|  | Carbon source | Heterotrophic |
| MIGS-6 | Habitat | artichoke, Rhizosphere |
| MIGS-6.3 | Salinity | 0–10% NaCl (w/v) |
| MIGS-22 | Oxygen requirement | Aerobic |
| MIGS-15 | Biotic relationship | Rhizospheric |
| MIGS-14 | Pathogenicity | Non-pathogen |
| MIGS-4 | Geographic location | Sidi Thabet, Tunisia |
| MIGS-5 | Sample collection | December, 2012 |
| MIGS-4.1 | Latitude | 36°54′31.1” N |
| MIGS-4.2 | Longitude | 10°2′32.89” E |
