# Supplementary material for: Pseudomonas rhizophila S211, a New Plant Growth-Promoting Rhizobacterium with Potential in Pesticide-Bioremediation
Source: Front Microbiol. 2018 Feb 23;9:34. doi: 10.3389/fmicb.2018.00034 (PMC5829100; doi:10.3389/fmicb.2018.00034)
Supplement: Supplementary file 2 [file Table2.DOC]

**Supplementary Table 2.** Genes related to plant growth promotion in S211 genome.

| **Plant growth promotion traits** | **Genes** | **Product name** | **Feature ID** |
| --- | --- | --- | --- |
| **Phosphate metabolism** |  | Alkaline phosphatase (EC 3.1.3.1) | fig|163011.11.peg.4088 |
| **Ammonia assimilation** | NaDPH-GOGAT | Glutamate synthase [NADPH] large chain (EC 1.4.1.13) | fig|163011.11.peg.1272 |
|  | GS | Glutamine synthetase type I (EC 6.3.1.2) | fig\|163011.11.peg.1527 |
|  | GOGATF | Ferredoxin-dependent glutamate synthase (EC 1.4.7.1) | fig\|163011.11.peg.2544 |
|  | glnK | Nitrogen regulatory protein P-II | fig\|163011.11.peg.72 |
| **Auxin biosynthesis** | trpA | Tryptophan synthase alpha chain (EC 4.2.1.20) | fig|163011.11.peg.1935 |
|  | trpB | Tryptophan synthase beta chain (EC 4.2.1.20) | fig\|163011.11.peg.1934 |
| **EPS synthesis** |  | Levansucrase (EC 2.4.1.10) | fig|163011.11.peg.2125 |
| **Siderophore Pyoverdine** | PvdA | L-ornithine 5-monooxygenase (EC 1.13.12.-), PvdA of pyoverdin biosynthesis | fig|163011.11.peg.3460 |
|  | PvdH | Pyoverdin biosynthesis protein, L-2,4-diaminobutyrate: 2-oxoglutarate aminotransferase (EC 2.6.1.76) | fig\|163011.11.peg.3358/ fig\|163011.11.peg.4916 |
|  | PvdM | Putative dipeptidase, pyoverdin biosynthesis | fig\|163011.11.peg.3452 |
|  | PvdN | Pyoverdin biosynthesis protein PvdN, putative aminotransferase, class V | fig|163011.11.peg.3451 |
| **Proline Synthesis** | P5CR (proC/ proG) | Pyrroline-5-carboxylate reductase (EC 1.5.1.2) | fig|163011.11.peg.223/ fig|163011.11.peg.4942 |
| **Denitrification** | NosZ | Nitrous-oxide reductase (EC 1.7.99.6) | fig|163011.11.peg.3950 |
|  | NirS | Cytochrome cd1 nitrite reductase (EC:1.7.2.1) | fig\|163011.11.peg.3965 |
| **Osmoregulation** | OmpA | Outer membrane protein A precursor | fig|163011.11.peg.861/ fig|163011.11.peg.862 |
|  | SOX | Sarcosine oxidase gamma subunit (EC 1.5.3.1) | fig\|163011.11.peg.1402 |
|  | ProX | L-proline glycine betaine binding ABC transporter protein ProX (TC 3.A.1.12.1) | fig\|163011.11.peg.1426/ fig\|163011.11.peg.1434/ fig\|163011.11.peg.1502/ fig\|163011.11.peg.1881/ fig\|163011.11.peg.4940 |
|  | OpuA | Glycine betaine/L-proline ABC transporter, glycine betaine/L-proline- binding/permease protein | fig\|163011.11.peg.2018 |
| **Cold shock** | CspA/ CspC/ CspD/ CspG | Cold shock protein | fig|163011.11.peg.2655/ fig|163011.11.peg.2619/ fig|163011.11.peg.1398/ fig|163011.11.peg.4973 |
| **Heat shock** | GrpE | Heat shock protein | fig|163011.11.peg.2243 |
|  | DnaJ/ DnaK | Chaperone protein | fig\|163011.11.peg.2245/ fig\|163011.11.peg.2244 |
