# Supplementary material for: Pseudomonas rhizophila S211, a New Plant Growth-Promoting Rhizobacterium with Potential in Pesticide-Bioremediation
Source: Front Microbiol. 2018 Feb 23;9:34. doi: 10.3389/fmicb.2018.00034 (PMC5829100; doi:10.3389/fmicb.2018.00034)
Supplement: Supplementary file 3 [file Table3.DOC]

**Supplementary Table 3.** ANOVA for the response surface quadratic model.

| **Responses** | **Source of variation** | **Sum of squares** | **Degrees of freedom** | **Mean square** | **Ratio** | **Significance** |
| --- | --- | --- | --- | --- | --- | --- |
| **Y1 : BS** **concentration (mg/L)** | Regression | 490152 | 20 | 24507.6 | 7.547 | ** |
|  | Residues | 32470.3 | 10 | 3247.03 |  |  |
|  | Validity | 28658.3 | 6 | 4776.38 | 5.011 | 7.2% |
|  | Error | 3812.03 | 4 | 953.007 |  |  |
|  | Total | 522622 | 30 |  |  |  |
| **: significant at the level of 99.9 %. | | | | | | |
