# Supplementary material for: Pseudomonas rhizophila S211, a New Plant Growth-Promoting Rhizobacterium with Potential in Pesticide-Bioremediation
Source: Front Microbiol. 2018 Feb 23;9:34. doi: 10.3389/fmicb.2018.00034 (PMC5829100; doi:10.3389/fmicb.2018.00034)
Supplement: Supplementary file 4 [file DataSheet1.DOC]

| 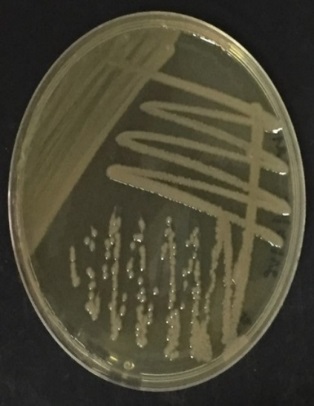 | | 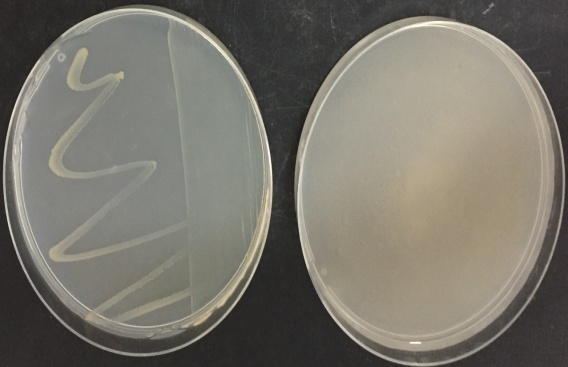 | | | | 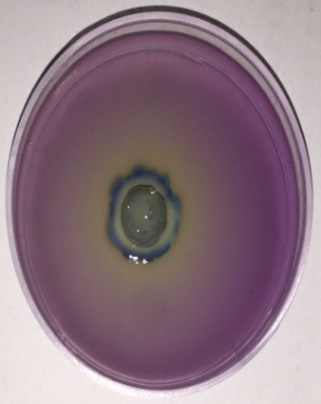 | | | **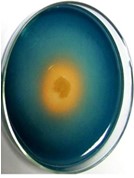** |
| --- | --- | --- | --- | --- | --- | --- | --- | --- | --- |
| **(a)** | | **(b)** | | | **(c)** | | | | **(d)** |
| 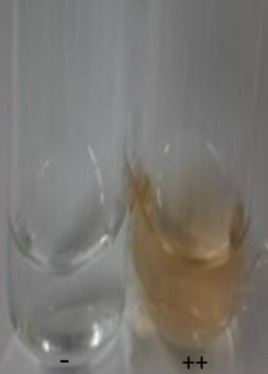 | | **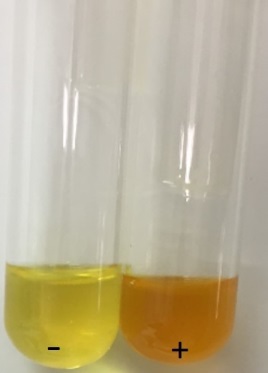** | | | | **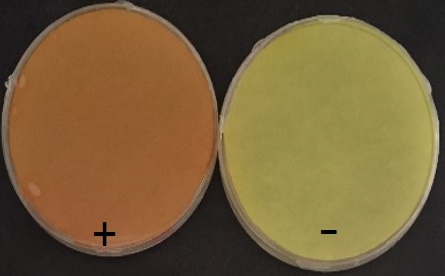** | | | |
| **(e)** | | **(f)** | | | **(g)** | | | | |
| **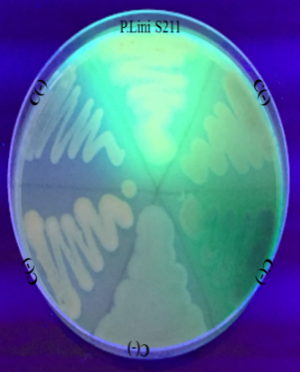** | | **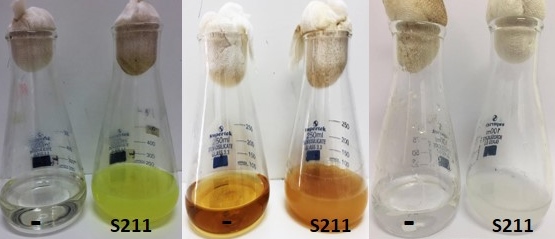** | | | | | | | **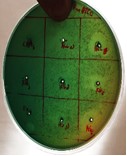** |
| **(h)** | | **(i)** | | | | | | | **(j)** |
| **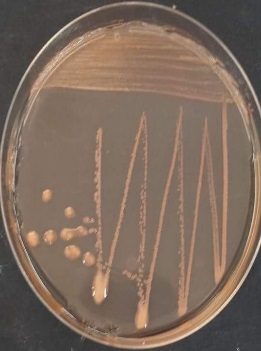** | **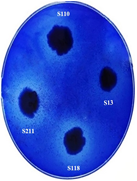** | | 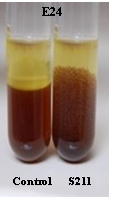 | | | | **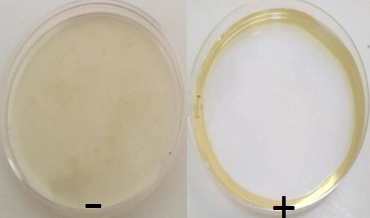** | | |
| **(k)** | | **(l)** | | **(m)** | | | | **(n)** | |

**Supplementary Figure 1.** Screening of *P. rhizophila* S211 strain for their multiple plant growth promoting activities**:** (a) Petri plate showing *P. rhizophila* S211 grown in NA medium: 10% NaCl, pH9. (b) N_2_-fixation. (c) Phosphorus solubilization. (d) Siderophore production. (e) IAA production. (f) NH3 production. (g) HCN production. (h) S211 in KB plate sous UV. (i) S211 liquid culture in CAA, KB and SM. (j) Bactericidal activity of pyoverdine extract. (k) EPS-producing potential. (l) Anionic BS detection in CTAB-MB medium. (m) Emulsification activity (E24). (n) Oil displacement activity of BS.
